# TF2LncRNA: Identifying Common Transcription Factors for a List of lncRNA Genes from ChIP-Seq Data

**DOI:** 10.1155/2014/317642

**Published:** 2014-03-04

**Authors:** Qinghua Jiang, Jixuan Wang, Yadong Wang, Rui Ma, Xiaoliang Wu, Yu Li

**Affiliations:** ^1^School of Life Science and Technology, Harbin Institute of Technology, Harbin, Heilongjiang 150001, China; ^2^School of Software, Harbin Institute of Technology, Harbin, Heilongjiang 150001, China; ^3^School of Computer Science and Technology, Harbin Institute of Technology, Harbin, Heilongjiang 150001, China

## Abstract

High-throughput genomic technologies like lncRNA microarray and RNA-Seq often generate a set of lncRNAs of interest, yet little is known about the transcriptional regulation of the set of lncRNA genes. Here, based on ChIP-Seq peak lists of transcription factors (TFs) from ENCODE and annotated human lncRNAs from GENCODE, we developed a web-based interface titled “TF2lncRNA,” where TF peaks from each ChIP-Seq experiment are crossed with the genomic coordinates of a set of input lncRNAs, to identify which TFs present a statistically significant number of binding sites (peaks) within the regulatory region of the input lncRNA genes. The input can be a set of coexpressed lncRNA genes or any other cluster of lncRNA genes. Users can thus infer which TFs are likely to be common transcription regulators of the set of lncRNAs. In addition, users can retrieve all lncRNAs potentially regulated by a specific TF in a specific cell line of interest or retrieve all TFs that have one or more binding sites in the regulatory region of a given lncRNA in the specific cell line. TF2LncRNA is an efficient and easy-to-use web-based tool.

## 1. Introduction

The Encyclopedia of DNA Elements (ENCODE) project has expanded our knowledge of what lies in the dark recesses of the human genome. One of these important findings is that only a small fraction of the human genome encodes proteins; almost 60% is represented in processed transcripts that seem to lack protein-coding capacity [1]. Long noncoding RNAs (lncRNAs) are non-protein-coding transcripts longer than 200 nucleotides in length. Compared to microRNAs or snoRNAs that exhibit strong conservation across diverse species, lncRNAs lack strong conservation, which is often cited as evidence of nonfunctionality [2, 3]. However, many well-described lncRNAs, such as Air and Xist, are poorly conserved [4], and increasing studies have demonstrated that a number of lncRNAs are not transcriptional noise but have important functions, such as regulating gene expression at various molecular levels, including protein, RNA, miRNA, and DNA [5–7].

Thousands of human lncRNAs have been identified [8], and accumulating studies have revealed that a number of lncRNAs play important roles in organismal development and various human diseases [9], such as cancers [10, 11], cardiovascular diseases [12], and neurodegeneration diseases [13]. However, few studies focus on how lncRNA genes themselves are transcriptionally regulated. Yang et al. developed a system by which users can browse transcription factor (TF) binding sites in the regulatory region of a lncRNA [14]. However, given a cell line or tissue of interest, users cannot obtain which transcription factors (TFs) have binding sites in the regulatory region of a specific lncRNA and which lncRNAs are regulated by a specific TF in the specific cell line or tissue.

Furthermore, high-throughput genomic technologies like lncRNA microarray (Arraystar Inc., Rockville, MD, USA) and RNA-Seq often generate a set of lncRNA genes of interest (e.g., coexpressed lncRNA genes). Given a set of lncRNA genes showing similar expression patterns, researchers often wonder how to find out which TFs are responsible for the observed expression pattern of the set of lncRNAs. For these kinds of problems, researchers used to examine whether the regulatory regions of the set of lncRNA genes contain an overrepresented sequence motif by using de novo sequence motif finding tools [15] or descriptors of the binding specificity of TFs, which may provide clues on which TFs could be common transcriptional regulators of the set of lncRNA genes. However, lncRNAs are temporally and spatially expressed and regulated, and motif-based sequence analysis cannot capture the dynamic regulation of lncRNAs by TFs in different cell lines.

Fortunately, chromatin immunoprecipitation followed by sequencing (ChIP-Seq) has enabled detecting transcription factor binding sites (TFBSs) with unprecedented sensitivity. The ENCODE project has completed ChIP-Seq experiments for many human TFs for a number type of cell lines. Enriched peak regions from the ChIP-Seq experiments of TFs can be crossed with the genomic coordinates of lncRNAs, which facilitate the discovery of TF-lncRNA regulatory relationships in a diversity of cell lines and also give us a better opportunity to identify common TFs for a given set of lncRNA genes in a cell line of interest.

Therefore, based on ChIP-Seq peak data from ENCODE and all annotated human lncRNAs from GENCODE, we developed a web-based tool titled “TF2LncRNA,” accessible at http://mlg.hit.edu.cn/tf2lncrna, which enables users to identify which TFs present a statistically significant number of peaks within the regulatory regions of a set of input lncRNA genes and thus identify common TFs that are likely to regulate the set of lncRNA genes. In addition, our tool enables researchers to easily browse and retrieve TF-lncRNA regulatory relationships for a specific TF or lncRNA in a specific cell line of interest.

## 2. Materials and Methods

### 2.1. Materials

#### 2.1.1. Genomic Annotations of lncRNAs

Genomic annotations of 13,249 human lncRNA genes and 22,531 lncRNA transcripts were downloaded from the GENCODE website (GENCODE version 15 that is identical to the Ensembl release 70) [16].

#### 2.1.2. Genome-Wide Binding Sites of TFs

Peak lists of 425 ChIP-Seq datasets performed on 148 TFs generated from uniform processing pipeline were downloaded from UCSC ENCODE Project Portal [1], where the PeakSeq [17] peak calling method was used to identify peaks (regions of enrichment) by comparing each ChIP-Seq experiment to a corresponding input DNA control experiment. Peak calling was performed independently on each replicate of a ChIP-Seq dataset, and a measure of consistency of peak calling results between replicates, known as the irreproducible discovery rate (IDR), was used to determine an optimal number of reproducible peaks.

### 2.2. Methods

#### 2.2.1. Associating Peaks of Transcription Factors with lncRNA Genes

A lncRNA gene was defined to be regulated by a TF, if the TF has at least one peak in the regulatory region of the lncRNA gene. Here, the regulatory region of a lncRNA gene is defined as a region that extends 2000 bp upstream and 1000 bp downstream from its transcription start site (denoted as −2 kb/+1 kb). We also considered other regulatory regions, such as −50 kb/+5 kb, −30 kb/+2 kb, −20 kb/+1 kb, and −10 kb/+1 kb.

#### 2.2.2. Finding Common Transcription Factors for a Set of lncRNA Genes Using Hypergeometric Test

The annotated human lncRNAs were downloaded from GENCODE website (version 15, i.e., Ensembl v70), which includes 13,249 annotated lncRNA genes and 22,531 lncRNA transcripts. Hypergeometric test (this method is usually applied to assess gene ontology or pathway enrichment for a list of protein-coding genes) is used to identify common TFs for a set of lncRNA genes or transcripts. For each ChIP-Seq experiment of a TF, a *P* value (i.e., a probability of obtaining *m* or more input lncRNA genes targeted by the TF by chance) is calculated by the formula below:

(1) $$\begin{array}{c} P=\frac{\sum_{i=m}^{\min\left(n,M\right)}\left(\begin{array}{c} M\\ i \end{array}\right)\left(\begin{array}{c} N-M\\ n-i \end{array}\right)}{\left(\begin{array}{c} N\\ n \end{array}\right)}, \end{array}$$

where

1. *N*  is the number of all annotated lncRNA genes (transcripts) in *Homo sapiens*;
2. *M* is the number of annotated lncRNA genes (transcripts) that contain at least one ChIP-Seq peak of the TF in their regulatory regions;
3. *n* is the number of lncRNA genes (transcripts) that users input;
4. *m* is the number of lncRNA genes (transcripts) in the inputted lncRNAs that have at least one peak of the TF within their regulatory regions.

All *P* values were adjusted by the Benjamini-Hochberg procedure for multiple testing corrections. A TF is defined as common TF of a set of lncRNAs if its adjusted *P* value is equal to or smaller than 0.05.

## 3. Web Interface

The web interface contains two panels on the left and right hand side, which allow users to input a set of lncRNAs for finding their common TFs or for browsing and retrieving TF-lncRNA regulatory relationships for a specific TF or lncRNA in a specific cell line of interest.

### 3.1. Browse and Retrieve TF-lncRNA Regulatory Relationships

**Figure 1:**
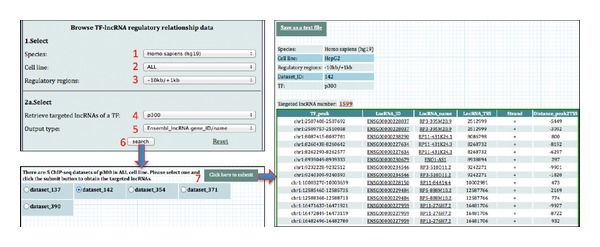
Browse and retrieve all lncRNAs potentially targeted by a specific TF in a specific cell line of interest.

**Figure 2:**
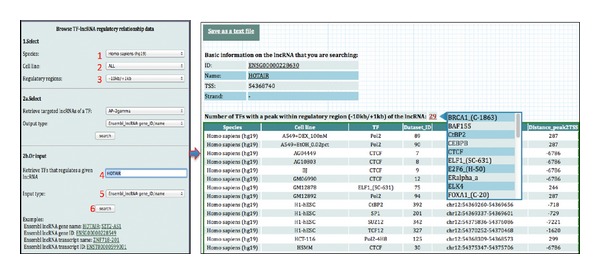
Browse and retrieve all TFs that have at least one peak in the regulatory region of specific lncRNA in various cell lines.

The right hand panel allows users to browse and retrieve TF-lncRNA regulatory relationships in a specific cell line of interest. Users can select (i) the source organism and the TF, (ii) the cell line in which the ChIP experiment was performed, (iii) the regulatory region of lncRNA genes (e.g., 2000 bp upstream and 1000 bp downstream from its TSS), and (iv) the lncRNA ID/name to be used to display the results. Therefore, given a TF of interest, users can retrieve all lncRNAs whose regulatory regions have at least one peak of the TF in the condition that users select (Figure 1). In addition, given a lncRNA of interest, users can also retrieve all TFs that have at least one peak in the regulatory region of the lncRNA in the condition that users design (Figure 2).

### 3.2. lncRNA Input

The left hand panel enables users to paste a set of lncRNA genes (the Ensembl lncRNA gene ID/name or lncRNA transcript ID/name) and to find TFs that have a significantly high number of peaks associated with the set of lncRNAs. Users then can select (i) the source organism of the lncRNA genes, (ii) the cell line in which the ChIP experiment was performed, (iii) the regulatory region, relative to the TSS of lncRNAs, and (iv) the input type, that is, what kind of lncRNA ID or name that users input, and, (v) optionally, users can upload a list of lncRNAs to define their own reference sets of lncRNAs. For example, if a lncRNA microarray study revealed x changing lncRNAs with a particular treatment, the reference set would not be all annotated human lncRNAs (default in TF2LncRNA system), but the user would provide a set of lncRNAs detected by the microarray to serve as the reference set or something similar.

### 3.3. Output

**Figure 3:**
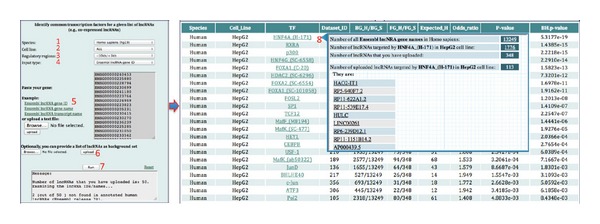
A schematic workflow of finding common TFs for a set of lncRNAs of interest in various cell lines.

After users input a set of lncRNAs of interest and upload a reference set (optionally), they click on the “Run” button. The system will first examine whether or not the input lncRNA IDs or names are correct and show the information in the message box and then identify common TFs based on the hypergeometric test. A schematic workflow is shown in Figure 3. From left to right the columns of the output result table summarize the following.

*Species*. From which species the transcription factor is.

*Cell Line*. In which cell line the ChIP-Seq experiment was performed.

*TF*. Transcription factor or other DNA-binding protein in the ChIP-Seq experiment.

*Dataset_ID*. An ID was assigned for each ChIP-Seq experiment.

*BG_H/BG_S*. Number of targeted lncRNA genes (transcripts) of the TF/number of all annotated lncRNA genes (transcripts) in *Homo sapiens*. If users upload a reference set, the BG_S will be the number of lncRNAs in the reference set.

*FG_H/FG_S*. Number of input lncRNA genes (transcripts) targeted by the TF/number of uploaded lncRNA genes (transcripts).

*Expected_H*. Expected number of lncRNA genes (transcripts) targeted by the TF within lncRNAs that users input and Expected_H = FG_S∗(BG_H/BG_S).

*Odds Ratio*. Ratio of the odds of lncRNAs targeted by a specific TF in your uploaded lncRNAs to the odds of lncRNAs targeted by a specific TF in all human lncRNAs and Odds_ratio = FG_H/(FG_S − FG_H)/BG_H/(BG_S − BG_H). 

*P Value*. Each *P* value is computed by the hypergeometric test.

*BH.P Value*. All *P* values are corrected by the Benjamini-Hochberg method.

## 4. Conclusion

We developed a web-based tool titled “TF2LncRNA” that enables researchers to easily find common transcription factors for a set of lncRNAs of interest, such as coexpressed lncRNAs. In addition, users conveniently browse and retrieve TF-lncRNA regulatory relationships for a specific TF or lncRNA gene in a specific cell line of interest. As the GENCODE annotations of lncRNAs will continue evolving and more ChIP-Seq data of TFs will become available, we will continue to maintain and improve TF2lncRNA as more data become available for facilitating the research on the transcriptional regulation of a set of lncRNAs.
